# The temporal mutational and immune tumour microenvironment remodelling of HER2-negative primary breast cancers

**DOI:** 10.1038/s41523-021-00282-0

**Published:** 2021-06-07

**Authors:** Leticia De Mattos-Arruda, Javier Cortes, Juan Blanco-Heredia, Daniel G. Tiezzi, Guillermo Villacampa, Samuel Gonçalves-Ribeiro, Laia Paré, Carla Anjos Souza, Vanesa Ortega, Stephen-John Sammut, Pol Cusco, Roberta Fasani, Suet-Feung Chin, Jose Perez-Garcia, Rodrigo Dienstmann, Paolo Nuciforo, Patricia Villagrasa, Isabel T. Rubio, Aleix Prat, Carlos Caldas

**Affiliations:** 1 IrsiCaixa, Germans Trias i Pujol University Hospital, Badalona, Spain; 2 Germans Trias i Pujol Research Institute (IGTP), Badalona, Spain; 3 Cancer Research UK Cambridge Institute, Robinson Way, Cambridge, UK; 4 Oncology Department International Breast Cancer Center (IBCC), Quiron Group, Barcelona, Spain; 5 Medica Scientia Innovation Research (MedSIR), Barcelona, Spain; 6 Medica Scientia Innovation Research (MedSIR), Ridgewood, NJ USA; 7 Breast Cancer Research program, Vall d´Hebron Institute of Oncology (VHIO), Barcelona, Spain; 8 Universidad Europea de Madrid, Faculty of Biomedical and Health Sciences, Department of Medicine, Madrid, Spain; 9 Breast Disease Division, Ribeirão Preto School of Medicine, University of São Paulo, São Paulo, Brazil; 10 Vall d’Hebron Institute of Oncology (VHIO), Vall d’Hebron University Hospital, Barcelona, Spain; 11 Department of Medical Oncology, Hospital Clinic of Barcelona, Barcelona, Spain; 12 SOLTI Breast Cancer Research Group, Barcelona, Spain; 13 Translational Genomics and Targeted Therapeutics in Solid Tumors, August Pi i Sunyer Biomedical Research Institute, Barcelona, Spain; 14 Department of Oncology, Cambridge University Hospitals NHS Foundation Trust, Cambridge, UK

**Keywords:** Cancer genomics, Breast cancer, Tumour biomarkers

## Abstract

The biology of breast cancer response to neoadjuvant therapy is underrepresented in the literature and provides a window-of-opportunity to explore the genomic and microenvironment modulation of tumours exposed to therapy. Here, we characterised the mutational, gene expression, pathway enrichment and tumour-infiltrating lymphocytes (TILs) dynamics across different timepoints of 35 HER2-negative primary breast cancer patients receiving neoadjuvant eribulin therapy (SOLTI-1007 NEOERIBULIN-NCT01669252). Whole-exome data (*N* = 88 samples) generated mutational profiles and candidate neoantigens and were analysed along with RNA-Nanostring 545-gene expression (*N* = 96 samples) and stromal TILs (*N* = 105 samples). Tumour mutation burden varied across patients at baseline but not across the sampling timepoints for each patient. Mutational signatures were not always conserved across tumours. There was a trend towards higher odds of response and less hazard to relapse when the percentage of subclonal mutations was low, suggesting that more homogenous tumours might have better responses to neoadjuvant therapy. Few driver mutations (5.1%) generated putative neoantigens. Mutation and neoantigen load were positively correlated (*R*^2^ = 0.94, *p* = <0.001); neoantigen load was weakly correlated with stromal TILs (*R*^2^ = 0.16, *p* = 0.02). An enrichment in pathways linked to immune infiltration and reduced programmed cell death expression were seen after 12 weeks of eribulin in good responders*. VEGF* was downregulated over time in the good responder group and *FABP5*, an inductor of epithelial mesenchymal transition (EMT), was upregulated in cases that recurred (*p* < 0.05). Mutational heterogeneity, subclonal architecture and the improvement of immune microenvironment along with remodelling of hypoxia and EMT may influence the response to neoadjuvant treatment.

## Introduction

Breast cancer is the most commonly diagnosed cancer and the leading cause of female cancer death worldwide^1^. It represents a heterogeneous group of tumours with characteristic molecular features, prognosis and responses to available therapy^2,3^.

In the early stage breast cancer setting, treatment decisions are guided by clinical subtypes, namely hormone receptor (HR) positive (HR+/HER2−), human epidermal growth factor receptor 2 amplified (HER2+) and triple-negative breast cancer (TNBC). This general classification does not take into account the complex genomic landscape and breast cancer evolution during therapy administration and disease recurrence or progression^4,5^.

Currently, the biology of the neoadjuvant response to therapy is underrepresented in the literature. Large-scale genomic studies have mostly focused on the analysis of single primary breast cancers^2,3,6–9^, which do not provide information on cancers over time. The analysis of the gene expression landscape of tumours has been shown to correlate with response to cytotoxic therapies^10–12^, though very limited work has been done to characterise the genomic and transcriptomic changes across breast cancer patients receiving neoadjuvant therapy^13^. Therefore, the neoadjuvant setting in breast cancer provides a window-of-opportunity to explore the genomic and microenvironment modulation of tumours exposed to therapy over time^5,14^.

In this study, we temporally characterised the mutational, gene expression, pathway enrichment and tumour-infiltrating lymphocytes (TILs) dynamics across different timepoints over a 12-week period in HER2-negative primary breast cancers enrolled in the single-arm SOLTI-1007 *NEOERIBULIN* phase II clinical trial (NCT01669252). We show that the mutational and immune tumour microenvironment remodelling of HER2-negative primary breast cancers provides a path forward for gathering biological insights from primary breast cancers.

## Results

### A clinical cohort of HER2-negative breast cancer patients

**Fig. 1:**
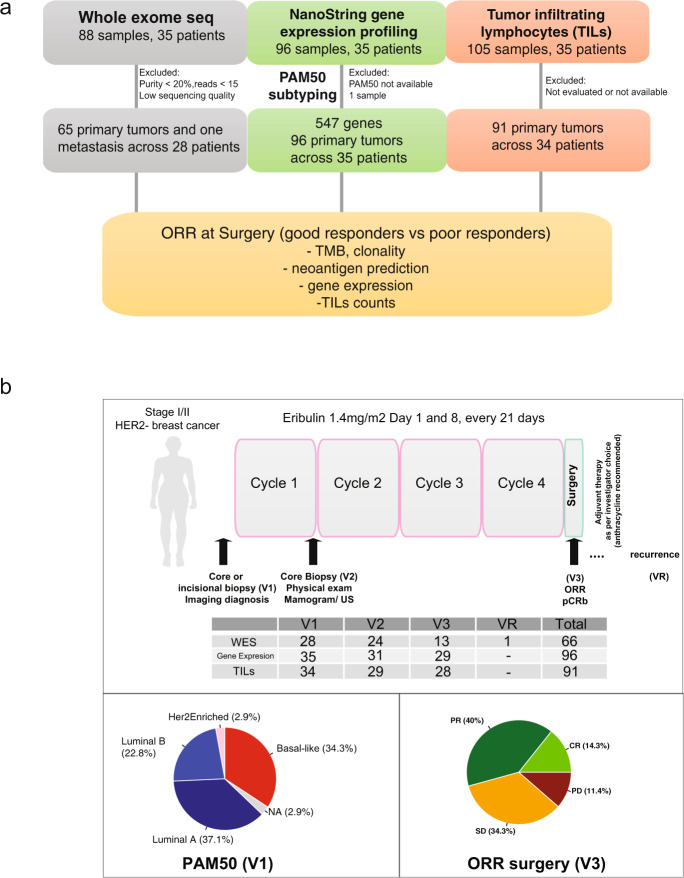
The study schematics. **a**. Tumour tissue samples underwent (i) Whole-exome sequencing (WES) for mutation and clonality detection followed by neoantigen prediction; (ii) Nanostring gene expression profiling; and (iii) stromal TILs counting. Our goal was to select samples that passed quality control and perform the temporal characterisation of the mutational, gene expression and TILs in serial primary HER2-negative breast cancers that were good responders or poor responders to eribulin. DNA sequencing (WES) was performed in 88 primary invasive breast cancers and matched the normal DNA of each patient. Of these, 66 tumour samples were used for mutational and clonality analyses. RNA-Nanostring gene expression profiling was performed in 96 primary invasive breast cancers. From the DNA sequencing data, candidate neoantigens were predicted. Stromal TILs were counted from the H&E slides in 91 out of 105 tumour specimens. Clinical features and the PAM50 intrinsic molecular subtypes of each of the sequential primary tumour’s biopsies were examined. TMB tumour mutation burden, ORR overall response rate. **b** Schematics of the clinical trial. Temporal tumour sampling and a number of samples included in each analysis and time point are depicted. Distribution of PAM50 molecular intrinsic breast cancer subtypes at V1 (diagnostic biopsy), and ORR at V3 (surgery). E eribulin administration, V1 visit one, V2 visit two, V3 visit three, VR visit recurrence, CR complete response, PR partial response, SD stable disease, PD progressive disease.

Primary breast cancer tumour specimens were obtained from the open-label, single-arm SOLTI-1007 *NEOERIBULIN* phase II clinical trial (NCT01669252). We included sequential primary tumour biopsies from 35 HER2-negative (22 HR-positive and 13 HR-negative) breast cancer patients (1–3 tumour tissue samples per patient) during eribulin administration. Whole-exome data (*N* = 88 samples) generated mutational profiles and candidate neoantigens and were analysed along with RNA-Nanostring 545-gene expression (*N* = 96 samples) and stromal TILs (*N* = 105 samples) from 35 patients with HER2-negative breast cancer (Fig. 1a).

Eighty per cent of cases had Ki67 greater than 14% at diagnosis. Disease recurrence after neoadjuvant therapy with eribulin was observed in six patients, despite the use of anthracyclines as adjuvant therapy. Although six patients presented clinic-radiologic recurrence, tumour material was available at the time of recurrence in one patient. The samples that passed quality control (i.e., tumour cellularity, sequencing quality, see Fig. 1a and “Methods”), were further processed and analysed.

Clinical features of the cohort are summarised in Table 1 and the schematics of the study design are shown in Fig. 1b. The 5-year relapse-free survival was 85.6% (95% CI: 74.7–98.1%) after breast cancer was diagnosed and the overall survival rate at 5 years was 91.3% (95% CI: 82.4–100%) for the patients analysed here.

**Table 1:** Clinicopathological characteristics of the study cohort.

Number of patients	35		*p* value (Fisher’s exact test)
Age (mean)	52.8 years		
Histologic grade	Diagnosis	Surgery	
Grade 1	4	1	
Grade 2	16	18	
Grade 3	14	14	0.5
Missing or not available	1	2	
*ER status*			
ER+	21	18	
ER−	14	15	0.8
Missing or not available	0	2	
*PR status*			
PR+	17	17	
PR−	18	16	1
Missing or not available	0	2	
*KI67 at diagnosis*			
> or = 14%	28	30	
<14%	7	3	0.3
Missing or not available	0	2	
*Clinical tumour stage at diagnosis*			
T0	0	2	
T1	1	21	
T2	31	10	
T3	2	1	<0.001
*Node*			
N0	24	19	
N1	11	12	
N2	0	4	0.1
*Histology*			
IDC	30	27	
ILC	2	2	
other	3	4	0.9
Missing or not available	–	2	
*Treatment response*			
pCR	2		
Good responders (CR or PR)	19 (5 CR, 14 PR)		
Poor responders (PR or SD)	16 (12 SD, 4 PD)		
Missing or not available	3		
Residual cancer burden (RCB) after neoadjuvant treatment	RCB1 = 1, RCB2 = 18, RCB3 = 13 patients, NA = 3		
Recurrence	6		

### The mutational and TILs profiling across temporal primary breast cancers

We characterised the mutational landscape of the tumours through an analysis of tumour mutational burden (TMB), breast cancer driver identification^15^, mutational signatures^16^ and mutational clonality^17^ at baseline (V1) (28 cases) and correlated these at visit 2 (V2) (24 cases) and with the response after 12 weeks of therapy at the time of surgery (V3) (13 cases).

**Fig. 2:**
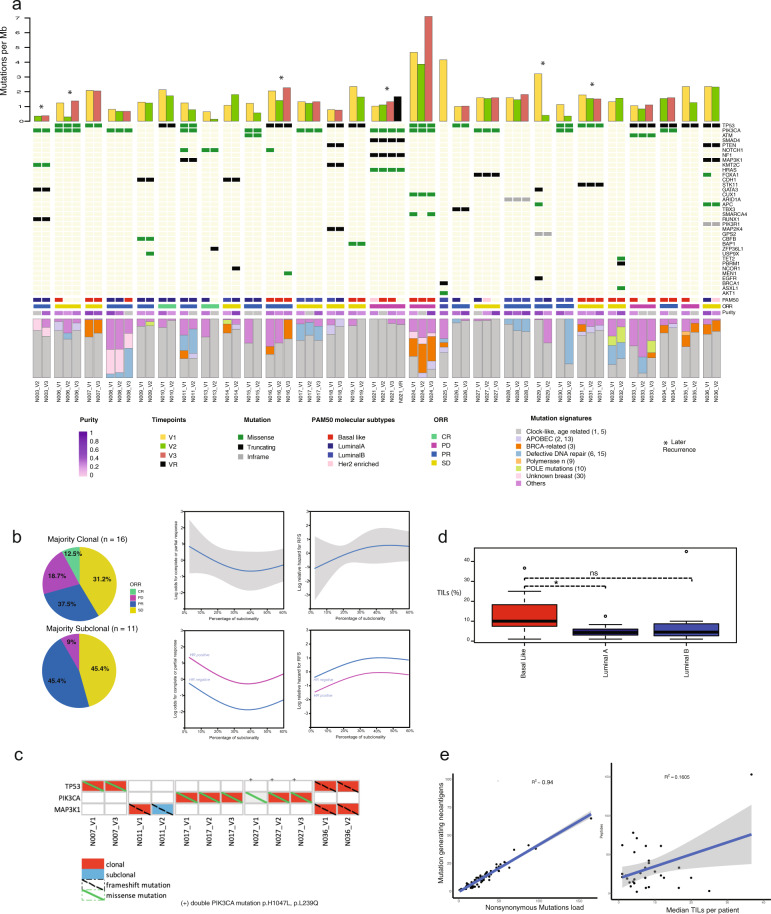
Mutational landscape of HER2-negative primary breast cancers under neoadjuvant chemotherapy. **a** Landscape of mutational alterations over time. Stacked plots display mutational burden (top), breast cancer drivers (tile plots, middle), PAM50 molecular intrinsic subtypes, clinical–pathological responses per patient and purity (tile plots, middle), mutation signatures (filled histogram). **b** Mutation clonality and subclonal distribution across different responses to eribulin. Left panels: odds for complete or partial response; right panels: a relative hazard for relapse-free survival (RFS). The analysis was performed for all comers (top panels) and for HR-positive and HR-negative patients (bottom panels). **c** Distribution of selected driver mutations generating neoantigens. Driver gene mutations are coloured whether the mutation is clonal or subclonal. **d** TILs across PAM50 intrinsic subtypes. (*) refer to *p* value < 0.05; ns nonsignificant. For each box plot, the centre line, the boundaries of the box, the ends of the whiskers and points beyond the whiskers represent the median value, the interquartile range, the minimum and maximum values, and the outliers, respectively. **e** Relationship between predicted neoantigen load (*y*-axis) and nonsynonymous mutational load (*x-*axis) and between predicted neoantigen load (*y*-axis) and mean stromal TILs per patient (*x*-axis).

TMB varied among patients at baseline (V1) (median 1.75 mutations per megabase (Mb) [range: 0.59–8.19], with a standard deviation of 1.48) but there was no evidence of variation across the three sampling time points (V2, median 1.62 mutations/Mb (range: 0.13–8.19), *p* = 0.62; V3, median 1.73 mutations/Mb (range: 0.38–7.11), *p* = 0.81) (mean coverage was ~40×) (Fig. 2a). At least one breast cancer driver gene^18,19^ was detected in 28 (80%) patients further analysed. *TP53* (*n* = 15) and *PIK3CA* (*n* = 11) were the most prevalent mutated driver genes in the cohort (Supplementary Fig. 1, Supplementary Data). Mutations within these breast cancer drivers were detected at all time points as well as in the recurrent sample (case N021). In V1, *TP53* mutation was more prevalent in basal cancers (*N* = 9), as compared to Luminal-A (*N* = 4) and Luminal-B (*N* = 2). Relapse free survival was not statistically significant when samples bearing *TP53* mutations in V1 (*N* = 15) were compared to those without the mutation (*N* = 20) [HR = 2.07, 95% CI: 0.35–12.4, *p* value = 0.43]. Likewise, no difference was found in patients with *PIK3CA* mutation (*n* = 11) [HR = 1.46, 95% CI: 0.24–8.75, *p* value = 0.68] (Supplementary Fig. 1). However, this cohort was not adequately powered to detect an association between mutations in driver genes and clinical outcomes.

We next investigated signatures of mutagenic biological processes to determine whether there were any detectable shifts during neoadjuvant therapy. A non-negative matrix factorisation technique to identify mutagenic processes in breast cancer including ageing, APOBEC cytidine deaminases, defective DNA repair, *BRCA1*/*BRCA2* deficiency was used^16^. Mutational signatures were studied in each tumour sample across neoadjuvant therapy (Fig. 2a—bottom panel). Signature 1 (C > T transitions at CpG dinucleotides) contributed to a relatively higher proportion of mutations, but others were predominant in some patients, such as APOBEC (signatures 2 and 13), were more prevalent in luminal B patients, and BRCA-related (signature 3) in basal-like tumours. Signatures were not always conserved across tumour samples (defective DNA repair signature was conserved in 62.5% of patients (*n* = 8) while APOBEC and BRCA-related in 40% (*n* = 7 and *n* = 10, respectively). No statistical differences were observed between mutation signatures and overall response rate (ORR) (all *p* values > 0.05, Fisher’s test) (Supplementary Fig. 1), although the study was not initially powered to detect this association.

We computed tumour clonal composition and CCFs by applying the ABSOLUTE^17^ algorithm across temporal primary tumours from each patient to determine how clonal composition relates to outcomes. Mutations predicted to be clonal (found in every cancer cell) or subclonal (found in a subset of cancer cells) were identified: the proportion of clonal mutations was 44.7% on the first visit, 21.0% on the second visit, 28.9% at surgery and 5% at recurrence).

We studied the shape of the association of subclonal mutations at baseline (V1) and the patientsʼ odds to obtain a complete or partial response (PR) and also the hazard to relapse (Fig. 2b, see “Methods”). Overall, there was a trend towards higher odds of response and less hazard to relapse when the percentage of subclonal mutations was low (i.e., from 0 to 40%), suggesting that less heterogeneous tumours might have better responses to neoadjuvant therapy. Further increases above 40% in the percentage of subclonal did not seem to impact outcomes. The same analysis was carried out for HR-positive and HR-negative patients (Fig. 2b, bottom panels). Overall, HR-positive patients had a trend to have better outcomes.

We generated HLA-class I binding predictions for somatic mutations that may result in protein changes. Totally, 492 out of 2009 (24.50%) unique mutations were predicted to encode for at least one strong binder (binding rank < 0.5%) of HLA-class I. We analysed candidate neoantigens generated from nonsynonymous mutations. A total of 1002 putative neoantigens (average of 36 per patient, [range: 2–153]) were present in 27 patients (Supplementary Data).

We inferred candidate neoantigens derived from breast cancer driver mutations (5.19% of total neoantigens) and whether they were clonal or subclonal. Overall, 52 neoantigens (average of 3.11 per patient [range: 1–11]) were generated by 22 (unique) driver mutations in 17 patients. Driver mutations including *TP53* (p.R110P, p.R209Kfs*6), *PIK3CA* (p.H1047L, p.K111E), *MAP3K1* (p.R961Sfs*44, p.E504Vfs*36) predicted neoantigens in more than one patient, and in general, they were clonal and present across all samples of each patient (Fig. 2c). Each candidate neoantigen was predicted to be bound to one or more HLA class-I molecule.

Finally, stromal TILs were counted in 91 samples of 34 patients and had a median of 9.3% [range: 0–40] among patients. Among the PAM50 intrinsic subtypes, basal-like tumours had significantly more TILs than luminal A (*p* value = 0.02) but not luminal B (*p* value = 0.11) (Fig. 2d, Supplementary Fig. 1).

We observed that the higher nonsynonymous mutation load was strongly correlated with neoantigen load and the latter was weakly correlated with stromal TILs. A significant positive correlation was observed in both analyses (*R*^2^ = 0.94, *p* = <0.001 and *R*^2^ = 0.16, *p* = 0.02), respectively (Fig. 2e).

Therefore, although TMB and driver mutations were temporally conserved across neoadjuvant therapy, cases with a lower percentage of subclonal mutations, thus more homogeneous breast cancers, had a trend for better responses. TILs were weakly correlated with neoantigen load, and a small fraction of neoantigens were derived from driver gene mutations.

### Multiple expressed genes across temporal primary breast cancers

To identify differentially expressed gene candidates during the administration of neoadjuvant chemotherapy, we performed targeted gene expression profiling using a custom 545-gene Nanostring panel composed of breast cancer-related genes among 35 patients (Supplementary Data).

**Fig. 3:**
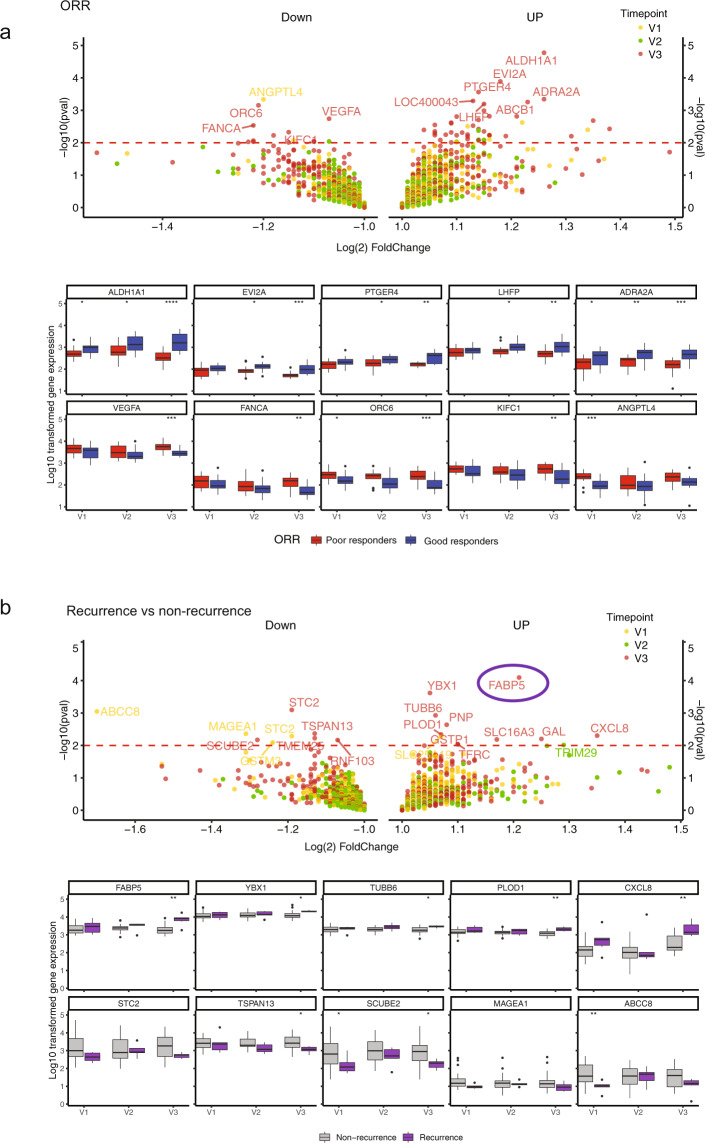
Gene expression profiling in longitudinal primary breast tumour biopsies. **a** ORR at surgery (V3) (patients with good response to eribulin vs. poor response) (Top). Genes identified have a change corresponding to eribulin treatment. Volcano plots with the strength of the association on the *y*-axis (−log10 *p* values) and the effect size on the *x*-axis (log 2-fold change (FC)). Differentially expressed genes were highlighted during different timepoints across neoadjuvant therapy (V1–V3). Genes above the red dotted line represent those whose expression levels were significantly different (*p* value < 0.01). A full list of the most up and downregulated genes can be found on Supplementary Data. Boxplots of good and poor responders on eribulin over time, colour-coded by their corresponding poor response (red) or good response (blue) in eribulin from baseline to surgery (Bottom). **b** Same as in (**a**). Patients with later clinical recurrence vs. no recurrence (Top). Boxplots of individual patients on eribulin over time, colour-coded by their corresponding recurrence (purple) or nonrecurrence (grey) in eribulin from baseline to surgery (Bottom).

We observed a total of 155 genes differentially expressed in patients responding to eribulin at surgery (good responders, CR or PR [*N* = 19]) vs. those with poor responses (PD or SD [*N* = 16]). After a false discovery rate (FDR) adjustment *p* value < 0.05, *ALDH1A1, EVI2A, ADRA2A, LHFP, LOC400043* and *PTGER4*, were upregulated while *VEGFA*, *FANCA, ORC6*, *KIFC1* and *ANGPTL4* were downregulated in the good responder group (Fig. 3a, Supplementary Data).

To assess variability within and between individuals, gene expression levels were analysed over a 12-week period for each patient (Fig. 3a bottom panels, Supplementary Fig. 2). Consistent with the general trend in the previous analysis, increased levels of *ALDH1A1, EVI2A, ADRA2A, LHFP, LOC400043* and *PTGER4* were modulated over time in patients responding to eribulin.

Overall and across all time points, we analysed patients that developed disease recurrence after curative therapy (*n* = 6) vs. those that did not recur (*n* = 29). We observed 58 upregulated genes in the group of patients with clinical recurrence as compared to those who did not have a recurrence. Among those were *FABP5*, *YBX1*, *TUBB6*, *PLOD1* and *CXCL8* genes that were upregulated and analysed over 12 weeks during neoadjuvant therapy.

Our analyses evidenced fatty acid-binding protein 5 (*FABP5*) overexpression at surgery (V3) was the only statically significant after FDR adjustment *p* < 0.05 in cases that recurred, as compared to those that did not recur (FDR = 0.04) (Fig. 3b, Supplementary Fig. 2). *FABP5* is an inductor of the epithelial-mesenchymal transition (EMT) transition process^20^. EMT has been reported to contribute to tumour aggressiveness and eribulin resistance^21,22^.

Finally, we used pathway enrichment analysis to identify the activity of the immune system and common cancer signalling pathways before and after neoadjuvant therapy (see “Methods”, Table 2). At the baseline V1 timepoint, it was observed an increase in pathways associated with angiogenesis, hypoxia and epithelial features, in patients that had poor responses to therapy (SD/PD). In the good responder group, eribulin treatment was associated with statistically significant enrichment of pathways associated to lymphocyte and T cell activation in the end of therapy (V3 or surgery), and with reduction in expression of the immunosuppressive programmed cell death pathway. In contrast, there was an increase in cell proliferation and reduction in cytotoxic T cell pathways for the poor responder group at V3 or surgery timepoint. This suggests that eribulin apparent reversal of EMT and restoration of hypoxia might lead to improvement of immunosuppressive features in the primary tumours of good responders, and an increase of proliferation and reduction of immune cytotoxic activity in poor responders.

**Table 2:** Gene ontology association with important biological pathways.

Visit	Effect in tested group (poor responders)	Pathway Database	Pathway	Genes	*p* value
V1	Increased	GO:0045766	Positive regulation of angiogenesis	PTGS2, VEGFA, AKT3, ADM, ANGPTL4 and CXCL8	0.00697
		KEGG:04066	HIF-1 signalling pathway	VEGFA, AKT3, ERBB2 and EGFR	0.0276
		GO:0050678	Regulation of epithelial cell proliferation	STRAP, VEGFA, AKT3, MYC, ERBB2, EGFR and NFIB	0.0217
		GO:1901342	Regulation of vasculature development	PTGS2, VEGFA, AKT3, ERBB2, ADM, ANGPTL4 and CXCL8	0.0462
V3	Decreased	GO:0046649	Lymphocyte activation	ERCC1, IGF1, IGBP1, CD86, IGFBP2, CDKN1A, VAV3, IL6ST, PIK3R1, ZEB1, TGFBR2, AXL, PTGER4 and KIF13B	0.0000413
		GO:0042110	T cell activation	IGF1, CD86, IGFBP2, IL6ST, PIK3R1, ZEB1, TGFBR2, PTGER4 and KIF13B	0.0227
		GO:0043069	Negative regulation of programmed cell death	IGF1, IGBP1, NR4A3, TWIST1, CDKN1A, IL6ST, KLF4, SLC40A1, CYR61, PIK3R1, BTG2, AXL and TWIST2	0.00716
		GO:0043066	Negative regulation of the apoptotic process	IGF1, IGBP1, NR4A3, TWIST1, CDKN1A, IL6ST, KLF4, SLC40A1, CYR61, PIK3R1, BTG2, AXL and TWIST2	0.00595
	Increased	GO:0012501	Programmed cell death	YBX3, GAL, MCM2, BIRC5, AARS, ITGA6, MYBL2, NDRG1, VEGFA, SNAI1, KRT17, TOP2A, CDK4, MYC, BRCA2, TP53BP2, CDCA7, HSPD1, ADM, CHEK1, MAD2L1, ANGPTL4, DDIT4, BUB1, CDK1, KRT19, KRT5, KRT14, S100A14 and PGAM5	0.00385
		GO:0008283	Cell proliferation	TACC3, STRAP, LAMC2, CDH3, GAL, GRHL2, BIRC5, CDC6, CDKN3, NDRG1, EZH2, FOXM1, BYSL, VEGFA, TTK, CDC20, CENPF, CKS2, DLGAP5, CCNB1, CDK4, MYC, BRCA2, KIF2C, CDCA7, HSPD1, CCNA2, MKI67, ADM, CHEK1, BTG3, CDC25C, FZD6, DDIT4, CXCL8, BUB1, CDK1, CTPS1, FANCA, PRC1 and BOP1	0.00000000266

## Discussion

Our study describes a step forward in gathering insights related to response to neoadjuvant therapy for HER2-negative primary breast cancers. Whole-exome sequencing, gene expression, pathway enrichment and TILs analyses of temporal primary breast tumours of 35 HER2-negative breast cancer patients show cancer remodelling during neoadjuvant chemotherapy.

The strengths of our analysis include the prospective design of the study, and the orthogonal genomic and immune infiltration data for each primary breast cancer systematically obtained from the SOLTI-1007 *NEOERIBULIN* phase II clinical trial (NCT01669252). Clonal driver mutations were maintained over time, but intratumoral genomic heterogeneity, measured as a fraction of subclonal mutations may affect response to therapy. Heterogeneity in mutational signatures across patients was considerably greater than at the intrapatient level, which was, in general, not conserved across temporal samples of each patient in 12 weeks of therapy administration. It should be noted, however, that eribulin is a weak chemotherapeutic agent which might explain the minimal change within the genomic landscape^21^. Its efficacy might be related to the fact that that it might change tumour phenotype (based on the EMT hypothesis)^21^ rather than changing tumour genotypes.

Neoantigens have been predicted in a few primary breast cancer datasets^23,24^. There is usually a discrepancy of neoantigens predicted computationally and those actually shown to leverage robust T-cell responses^25,26^. An immunosuppressive tumour microenvironment might be associated with this fact. A recent analysis of The Cancer Genome Atlas (TCGA) dataset of invasive breast cancers, predicted HLA class I-binding neoepitopes for 870 breast cancer samples^24^. About 40% of the nonsynonymous mutations led to the generation of candidate neoepitopes and the neoepitope load was also highly correlated with the mutational burden. Here, 21% of the nonsynonymous mutations led to the generation of putative neoantigens. Candidate neoantigens and stromal TILs were positively correlated and were more frequent in basal-like primary tumours. Driver mutations such as frameshifts mutations in *TP53* and *MAP3K1* genes or missense mutations in the *PIK3CA* gene can generate tumour neoantigens, suggesting that a T cell-mediated immune response, if present and properly validated, would target all cancer cells^15,27,28^. The relevance of neoantigens derived from clonal driver mutations, particularly those arising from truncating mutations, is the possibility of being incorporated as targets for adoptive T cell therapies and cancer vaccines^28,29^.

The evaluation of TILs has been shown to represent a reliable surrogate of the immune anti-tumour activity and a robust independent prognostic biomarker in breast cancer patients, especially in the TNBC and HER2-positive breast cancer subtypes^30,31^. Retrospective reports analysed TILs as prognostic and predictive markers in metastatic TNBC patients that received eribulin^32^. In a previous analysis, the high TILs group vs low TILs within the metastatic TNBC cases had better outcomes and suggested TILs to predict treatment response to eribulin in the TNBC metastatic setting^32^. In our early stage breast cancer cohort, stromal TILs detected in H&E slides were more frequent in basal-like primary tumours. A statistically significant higher percentage of stromal TILs after the end of neoadjuvant therapy was not observed possibly due to the small sample size and other biological factors including immunosuppressive TME. However, we observed an enrichment in pathways associated with immune infiltration and reduced programmed cell death after 12 weeks of eribulin administration corroborating findings observed in metastatic breast cancer. In a recent study^33^, lymphocyte infiltration increase and PDL1 expression turning to negative values were suggested to be improvements in the immunosuppressed TME of patients receiving eribulin for advanced breast cancer. Our findings add to current evidence for potentially combining checkpoint inhibitors to eribulin in the early breast cancer setting^34^.

There is the emerging rationale for eribulin to activate the immune system in pre-clinical and retrospective studies, through EMT suppression, and vascular remodelling and improvement of the tumour immune microenvironment^21^. Our analyses indicate that temporal gene expression responses to eribulin might be associated with the process of restoration of hypoxia, and the EMT process, which should be contextualised with the immune TME. Eribulin treatment has shown to suppress genes that are known to be involved in hypoxic signalling cascades, including VEGF, contributing to TME remodelling and restoring the scenario of normoxia^35,36^. We observed that *VEGF* was downregulated overtime during eribulin therapy in the good responder group. At diagnosis, poor responders presented an overexpression of angiopoietin-like 4 (*ANGPTL4)*, a pro-angiogenic factor that is modulated by hypoxia and associated with poor prognosis, metastasis, cell differentiation and vascular permeability^37^. Previously, aldehyde dehydrogenase 1A1 (*ALDH1A1*) was reported as a cancer stem cell marker and suggested to have a favourable prognostic role for cervical cancer^38,39^. Although we observed *ALDH1A* to be upregulated in good responders, its role in breast cancer is undefined yet. Furthermore, *FABP5* promotes tumour cell growth in numerous cell types and is a negative prognostic marker in renal cell carcinoma^20,40^. *FABP5* was upregulated in cases that presented further clinic-radiological recurrence. However, identification of *FABP5* as a biomarker in HER2-negative breast cancer requires substantially increased cohort size and mechanistic validation for robust interpretation.

Our work has limitations which are mostly secondary to the small sample size and therefore lack of power to detect specific associations. Nonetheless, this HER2-negative breast cancer cohort gives a glimpse of the changes in cancer biology during neoadjuvant therapy administration. Another limitation is the 545 targeted gene expression panel used did contemplate only a few immune markers including *IDO1*, *LAG3* and *STAT1*. Furthermore, the primary endpoint of the present clinical trial is pathologic complete response (pCR) in the breast. We used ORR for analyses of responses after the neoadjuvant therapy since there are only a few pCR cases; residual cancer burden was used but was not informative in the dataset; thus, the association between biomarkers and response is challenging.

Taken together, these results suggest that mutational heterogeneity, subclonal architecture and the immune microenvironment along with remodelling of hypoxia and EMT may influence the response to neoadjuvant treatment in early stage disease, with possible implications for clinical decision-making and monitoring of treatment efficacy.

## Methods

### Patients and tissues

Primary breast cancer tumour specimens were obtained from the phase II study, open-label, single-arm SOLTI-1007 *NEOERIBULIN* (NCT01669252). The patients reported here were treated at the Vall Hebron Institute of Oncology, Barcelona. In brief, eribulin was administered as neoadjuvant treatment for stage I-II HER2-negative breast cancer with a dose of 1.4 mg/m^2^ intravenously on Days 1 and 8 every 21-day cycle, for 4 cycles, and the pathological CR was defined as the absence of residual invasive tumour in breast tumour specimens. All patients were negative for HER2 overexpression on clinical assays. The Ethics Committee of the Vall Hebron University Hospital, Barcelona, Spain approved the study. All patients gave informed consent for DNA and RNA sequencing.

### Clinic-pathological response analyses

We adopted a conservative method of defining ORR after neoadjuvant therapy as CR or PR by response evaluation criteria in solid tumours (RECIST) (major decrease in tumour burden following treatment) and poor response as progressive disease (PD) or stable disease (SD) by RECIST (major increase or stability in tumour burden following treatment) after eribulin therapy.

Patients were classified into two groups depending on their clinical response to therapy at surgery: *good responders*—patients that had CR or PR; *poor responders*—patients that had PD or SD.

### Tissue processing and DNA and RNA extraction

Frozen biopsies were embedded in a frozen tissue matrix (OCT; Sakura Finetek, Torrance, CA) and cut at the cryostat for tumour cellularity assessment by a pathologist. Genomic DNA was isolated from 10 × 10um sections using the DNeasy Kit (Qiagen, Hilden, Germany). Samples with less than 10% tumour material or that produced low DNA yield were excluded from the analysis.

A section of the FFPE breast tissue was first examined with haematoxylin and eosin staining to confirm the presence of invasive tumour cells (≥10%).

For RNA purification (High Pure Formalin-Fixed Paraffin-embedded RNA Isolation Kit, Roche Diagnostics Limited, West Sussex, UK), one to five 10 μm FFPE slides were used for each tumour specimen (at diagnosis, cycle 2 and surgery).

### Gene expression and Intrinsic subtype analyses

A minimum of ∼125 ng of total RNA was used to measure the expression of 545 genes involved in breast cancer, including 5 housekeeping genes (*ACTB*, *MRPL19*, *PSMC4*, *RPLP0* and *SF3A1*), using the Prosigna assay (NanoString Technologies, Seattle, USA). Samples with 20 or fewer counts in at least 70% of the genes were removed. Data were log base 2 transformed and normalised using the housekeeping genes.

The same RNA was used to measure the expression of 50 genes of the PAM50 intrinsic subtype predictor assay. For each sample, we calculated the PAM50 signature scores (Basal-like, HER2-E, Luminal A and B) and the proliferation signature score^41^. Differential gene expression analysis fold change of each gene was calculated as the ratio of average gene expression intensity of (i) the good responder group (*n* = 19 [5 CR, 14 PR]) to that of the poor responder group (*n* = 16 [12 SD, 4 PD]) or (ii) the patients that recurred (*n* = 6) to that of the non-recurrent group (*n* = 29). A two-sample *t* test was used to compare gene expression intensities between groups.

A gene was claimed to be differentially expressed if it showed a fold change of >1 (increased in good responders, or non-recurrent) or ≤ −1 (increased in poor responders, or in the later recurred) and further adjustment FDR ≤ 0.05 was applied. Volcano plots were used to visualise log 2-fold change on the *x*-axis and −log10 *p* values on the *y*-axis.

Pathway enrichment analysis was performed using the gprofiler toolkit^42^ comparing good responder vs. poor responder groups using two ontology databases as reference: (i) Gene Ontology and (ii) Kyoto Encyclopaedia of Genes and Genomes.

### DNA sequencing

WES was performed to the breast cancer tumour and matched normal DNA obtained from the buffy coat of each HER2-negative breast cancer patient.

Libraries for Illumina sequencing were prepared using Illumina Nextera Rapid Capture Exome kit (cat. FC-140-1003, Illumina) as we reported previously. Prior to library preparation DNA concentrations for each sample were quantified using a fluorescence-based method (Quant-IT dsDNA BR, cat. Q33130, Thermo Fisher Scientific) and 50 ng of genomic DNA was used for library preparation.

Samples were processed following the manufacturer’s instructions (part# 15037436 Rev. J, Illumina) for WES. Prior to the first hybridisation, all libraries were quantified using quantitative polymerase chain reaction (qPCR). KAPA Library Quantification Kit (cat. KK4873, KAPA Biosystems) as used as per manufacturer’s recommendations. A subset of libraries was analysed using DNA 1000 Kit (cat. 5067-1504, Agilent).

Whole-genome libraries and exome libraries were normalised and pooled in equal volumes to create balanced pools. Each pool was normalised to molarity of 4 nM and used for sequencing with clustering concentration 20 pM with 1% spike-in of PhiX control. Sequencing was performed on an Illumina HiSeq2500 using v4 chemistry and 50 cycles single-end for s-WGS and 75 cycles paired-end for WES. Demultiplexing was performed using Illumina’s bcl2fastq2 v.2.17 software using default options. FASTQ files were used for subsequent data analysis.

### WES analyses

For WES analysis, reads were mapped to the human genome (GRCh37) and base quality recalibration were performed using Novoalign v 3.02 (Novocraft). Coordinate sorting of reads and PCR-duplicate marking was performed using Novosort (v 3.02). The resulting bam files for all samples for the same patient were locally realigned using the Genome Analysis Toolkit (GATK, v 3.4.46)^43^. MuTect (version 2) was run using default parameters^44^. Strelka (version 1.0.14) was run with recommended starting parameters for BWA and default parameters^45^. The mean coverage per sample was calculated with CollectWgsMetrics (Picard). A joint calling was done and filtered allelic fraction >10% in at least one sample of each patient. Somatic mutations were annotated using Variant Effect Predictor (VEP, http://grch37.ensembl.org/) and visualised using IGV. Output with reads > 15 and purity > 20% were included in further analyses.

### ABSOLUTE

ABSOLUTE (v1.0.6) was used to infer the cancer cell fraction (CCF) of mutations and the mutations were classified as clonal or subclonal as previously described^17,46^. A mutation was classified as clonal if its probability of being clonal was >50% or if the lower bound of the 95% confidence interval of its CCF was >90%. Mutations that did not meet the above criteria were considered subclonal.

ABSOLUTE software was used to calculate tumour clonality, purity and ploidy. For running the ABSOLUTE we obtained the mutation annotation file by running vcf2maf script with VCF files for the corresponding tumour and normal control samples, annotation was performed by VEP. To find the segmentation we ran the CNVkit batch command with the.bam files from tumour and normal samples^47^. For running ABSOLUTE we subset the.cns files from the CNVkit output file for variant coordinates as well as probes and log2 values. We used the options min.ploidy = 0.95, max.ploidy = 10. Probability of a mutation to be clonal was defined essentially as described in ref. ^48^.

### Mutation signature

Decomposition of the mutational signature was performed using deconstructSigs^16^, based on the set of 30 mutational signatures. These signatures are largely defined by the relative frequency of the six possible base substitutions (C > A, C > G, C > T, T > A, T > C, and T > G) in the sequence context of their adjacent 5′ and 3′ base. Of these, COSMIC signatures 1, 2, 3, 6, 13, 9, 10, 15, have been associated with breast cancer. For clarity, we explored the following mutational signatures in this cohort: ageing/clock-like signatures^49,50^ (signatures 1 and 5), APOBEC (signatures 2 and 13), BRCA-related (signature 3), defective DNA repair (signatures 6 and 15), polymerase n (signature 9), POLE mutations (signature 10), unknown breast (signature 30) and other signatures.

### Neoantigen prediction

The 4-digit HLA type was obtained from matched normal WES data and the OptiType 1.3.1 analysis software package was used^51^. WES and HLA typing was integrated, and NeoPredPipe pipeline^52^ was applied with minor modifications for the neoantigen prediction. First, non-synonymous cancer-specific mutations, i.e., present in all tumour cells and absent in all normal cells, were used to generate a comprehensive list of peptides (9–11 amino acids in length) with the mutated amino acid represented at each peptide position and used as input was previous described. *Prioritisation of neoantigens*: the selection of candidate neoantigens were based on a %Rank < 0.5; epitopes already existing in the reference proteome indicated by the Novelty parameter were excluded.

### Tumour infiltrating lymphocytes

Evaluations of stromal TILs were performed on haematoxylin and eosin-stained sections by an experienced board-certified pathologist (R.F.) according to the 2014 recommendations of the international TILs working group^53^.

### Statistical analysis

Continuous variables were expressed as median and range, while categorical variables were expressed as absolute values or percentages. For statistical comparison, we used Mann–Whitney test for independent continuous variables, Wilcoxon signed-rank test for paired continuous data and Fisher’s exact test for categorical data. To study if there was a significant variation of TMB across sampling timepoints the Wilcoxon signed-rank test was used to compare pairwise timepoints. In addition, the range and standard deviation of TMB in each time point were calculated to estimate intra-period TMB heterogeneity. To study the association between mutations in driver genes and relapse-free survival the Cox proportional hazard model was fitted. Furthermore, to analyse the association between the percentage of subclonal mutations at baseline and clinical outcomes, we used the logistic and Cox models after relaxed linearity assumption using restricted cubic splines by means of *rms* R package. Data analyses were carried out using R version statistical software 3.6.3.

### Reporting summary

Further information on research design is available in the Nature Research Reporting Summary linked to this article.

## Supplementary information

Supplementary Data 1

Supplementary Information

Reporting Summary

## Data Availability

The data generated and analysed during this study are described in the following data record: 10.6084/m9.figshare.14454261^54^. The whole-exome sequencing bam files have been deposited at the *European Genome-phenome Archive* (EGA), which is hosted by the EBI and the CRG, under the study accession number https://identifiers.org/ega.study:EGAS00001004953 and dataset accession number https://identifiers.org/ega.dataset:EGAD00001006980^55^. The decreased and increased pathway enrichment analyses are available via GitHub at https://github.com/NeoVaCan/NPJBCANCER_DeMattos_2021/tree/main/Data_Supp_Table2. The supplementary tables are also available in Excel format as part of the *figshare* data record. The patient metadata and patient tumour-infiltrating lymphocytes data are not publicly available for the following reason: data contain information that could compromise research participant privacy. However, the data can be made available upon reasonable request to the corresponding author.

## Code availability

Custom R code used to analyse tumour whole-exome sequencing data and Nanostring gene expression data are available at https://github.com/NeoVaCan/NPJBCANCER_DeMattos_2021.
