# Supplementary material for: The temporal mutational and immune tumour microenvironment remodelling of HER2-negative primary breast cancers
Source: NPJ Breast Cancer. 2021 Jun 7;7:73. doi: 10.1038/s41523-021-00282-0 (PMC8185105; doi:10.1038/s41523-021-00282-0)
Supplement: Supplementary file 2 — Supplementary Information [file 41523_2021_282_MOESM2_ESM.pdf]

Supplementary Information

The temporal mutational and immune tumour microenvironment remodelling of HER2-negative primary breast cancers

Leticia De Mattos-Arruda et al.

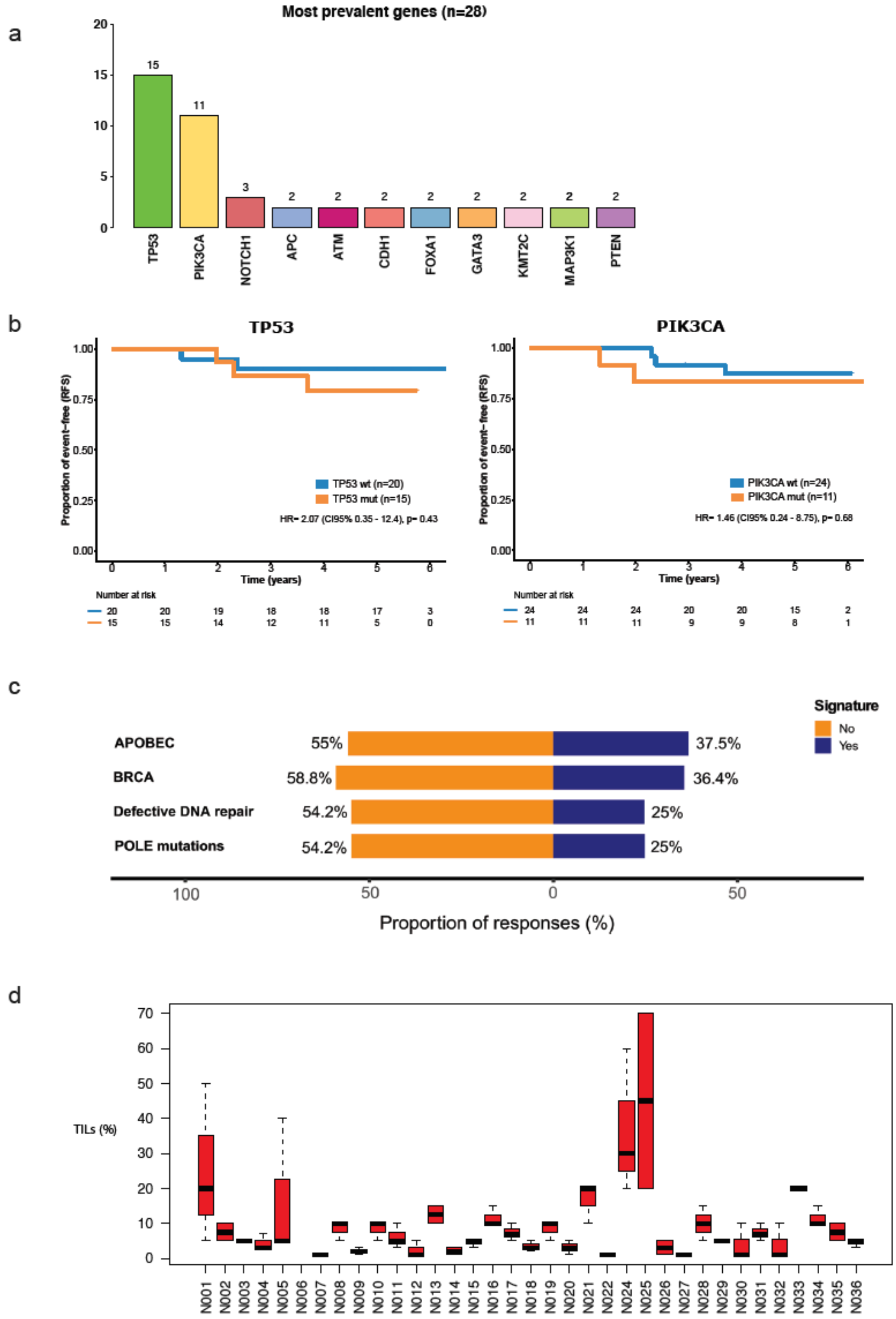

Supplementary Figure 1. a Prevalent driver gene mutations across all patients. **b**. Kaplan–Meier Relapse Free Survival curves for *TP53* and *PIK3CA* mutations. **c.** Distribution of mutation signatures among good responders to eribulin. **d.** Distribution of TILs across patients. For each box plot, the center line, the boundaries of the box, the ends of the whiskers and points beyond the whiskers represent the median value, the interquartile range, the minimum and maximum values, and the outliers, respectively.

a

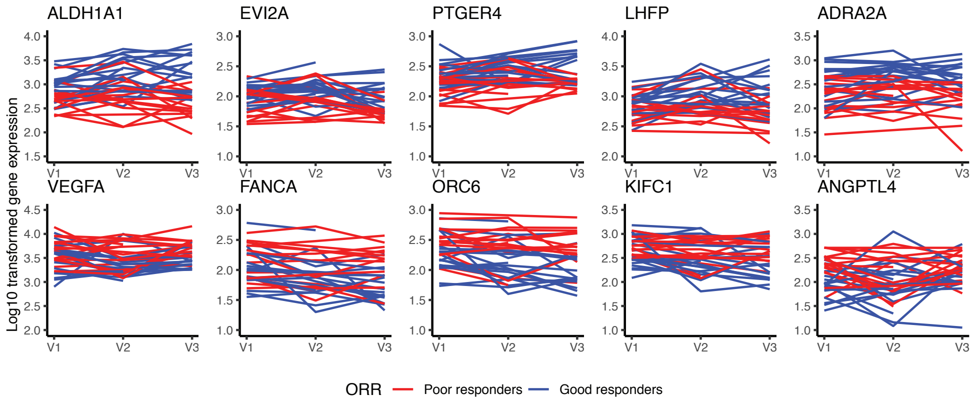

b

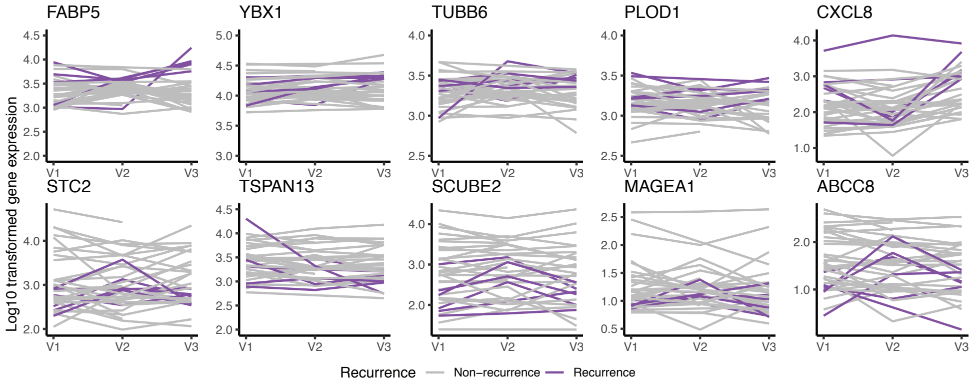

Supplementary Figure 2.

a. Trajectories of individual patients on therapy over time, colour-coded by their corresponding poor response (red) or good response (blue) from baseline to surgery. **b.** Trajectories of individual patients on therapy over time, colour-coded by their corresponding recurrence (purple) or nonrecurrence (grey) from baseline to surgery.

Supplementary Tables

Supplementary Table 1. Distribution of mutations across samples.

Supplementary Table 2. Predicted neoantigens across samples.

Supplementary Table 3. Nanostring gene expression normalized data.

Supplementary Table 4. Upregulated genes across patients treated with eribulin.

Supplementary Table 5. Downregulated genes across patients treated with eribulin.
