# Supplementary material for: The temporal mutational and immune tumour microenvironment remodelling of HER2-negative primary breast cancers
Source: NPJ Breast Cancer. 2021 Jun 7;7:73. doi: 10.1038/s41523-021-00282-0 (PMC8185105; doi:10.1038/s41523-021-00282-0)
Supplement: Supplementary file 3 — Reporting Summary [file 41523_2021_282_MOESM3_ESM.pdf]

nature research

Corresponding author(s): NPJBCANCER-00938R1 L. De Mattos-Arruda

Last updated by author(s): Mar 25, 2021

Reporting Summary

Nature Research wishes to improve the reproducibility of the work that we publish. This form provides structure for consistency and transparency in reporting. For further information on Nature Research policies, see our Editorial Policies and the Editorial Policy Checklist.

Statistics

For all statistical analyses, confirm that the following items are present in the figure legend, table legend, main text, or Methods section.

n/a	Confirmed	
☐	☒	The exact sample size (n) for each experimental group/condition, given as a discrete number and unit of measurement
☒	☐	A statement on whether measurements were taken from distinct samples or whether the same sample was measured repeatedly
☐	☒	The statistical test(s) used AND whether they are one- or two-sided<br>*Only common tests should be described solely by name; describe more complex techniques in the Methods section.*
☐	☒	A description of all covariates tested
☐	☒	A description of any assumptions or corrections, such as tests of normality and adjustment for multiple comparisons
☐	☒	A full description of the statistical parameters including central tendency (e.g. means) or other basic estimates (e.g. regression coefficient) AND variation (e.g. standard deviation) or associated estimates of uncertainty (e.g. confidence intervals)
☐	☒	For null hypothesis testing, the test statistic (e.g. F, t, r) with confidence intervals, effect sizes, degrees of freedom and P value noted<br>*Give P values as exact values whenever suitable.*
☒	☐	For Bayesian analysis, information on the choice of priors and Markov chain Monte Carlo settings
☒	☐	For hierarchical and complex designs, identification of the appropriate level for tests and full reporting of outcomes
☐	☒	Estimates of effect sizes (e.g. Cohen's d, Pearson's r), indicating how they were calculated

Our web collection on statistics for biologists contains articles on many of the points above.

Software and code

Policy information about availability of computer code

Data collection	No software was used for data collection

Data analysis

Data analysis:

Demultiplexing: Illumina's bcl2fastq2 v.2.17 software
Mapping: Novoalign v 3.02 (Novocraft) to the reference GRCh37
Sorting: Novosort v 3.02 (Novocraft)
Realignment: Genome Analysis Toolkit (GATK, v 3.4.46)
Mutation calling: MuTect v2
Small variant calling: Strelka v 1.0.14
Alignment: BWA v0.7.11
Mean coverage calculation: CollectWgsMetrics by Picard v4.0
Mutation annotation: VEP release 87
Visualization: IGV v2.4
Cancer Cell Fraction: ABSOLUTE v1.0.6
Segmentation: CNVkit v0.9.0
deconstructSigs: R library
HLA typing OptiType 1.3.1
Neoantigen prediction: NeoPredPipe 1.1
Statistical analysis: R 3.6.3
Libraries "cowplot", "dplyr", "ggplot2", "ggrepel" and "rms".

For manuscripts utilizing custom algorithms or software that are central to the research but not yet described in published literature, software must be made available to editors and reviewers. We strongly encourage code deposition in a community repository (e.g. GitHub). See the Nature Research guidelines for submitting code & software for further information.

Data

Policy information about availability of data

All manuscripts must include a data availability statement. This statement should provide the following information, where applicable:

- Accession codes, unique identifiers, or web links for publicly available datasets
- A list of figures that have associated raw data
- A description of any restrictions on data availability

Data Availability: The whole exome sequencing bam files are been deposited at the European Genome-phenome Archive (EGA), which is hosted by the EBI and the CRG, under the accession number EGAS00001004953.

Code availability: Custom R code used to analyze tumor whole exome sequencing data and Nanostring gene expression data is available at https://github.com/NeoVaCan/NPJBCANCER_DeMattos_2021

Field-specific reporting

Please select the one below that is the best fit for your research. If you are not sure, read the appropriate sections before making your selection.

☒ Life sciences ☐ Behavioural & social sciences ☐ Ecological, evolutionary & environmental sciences

For a reference copy of the document with all sections, see nature.com/documents/nr-reporting-summary-flat.pdf

Life sciences study design

All studies must disclose on these points even when the disclosure is negative.

Sample size	35 patients
Data exclusions	Whole Exome sequencing data: Output with reads < 15 and purity <20% were excluded in further analyses.<br>Nanostring gene expression profiling: Samples with 20 or fewer counts in at least 70% of the genes were removed.
Replication	n/a
Randomization	n/a
Blinding	n/a

Reporting for specific materials, systems and methods

We require information from authors about some types of materials, experimental systems and methods used in many studies. Here, indicate whether each material, system or method listed is relevant to your study. If you are not sure if a list item applies to your research, read the appropriate section before selecting a response.

Materials & experimental systems

n/a	Involved in the study	
☒	☐	Antibodies
☒	☐	Eukaryotic cell lines
☒	☐	Palaeontology and archaeology
☒	☐	Animals and other organisms
☐	☒	Human research participants
☐	☒	Clinical data
☒	☐	Dual use research of concern

Methods

n/a	Involved in the study	
☒	☐	ChIP-seq
☒	☐	Flow cytometry
☒	☐	MRI-based neuroimaging

Human research participants

Policy information about studies involving human research participants

Population characteristics	The stage I-II HER2-negative breast cancer patients reported here were treated at the Vall Hebron Institute of Oncology, Barcelona during the years 2012 and 2013 in the open-label, single-arm SOLTI-1007 NEOERIBULIN phase II clinical trial (NCT01669252).
Recruitment	Enrolled patients in the open-label, single-arm SOLTI-1007 NEOERIBULIN phase II clinical trial (NCT01669252) were included in this study.
Ethics oversight	The Ethics Committee of the Vall Hebron University Hospital, Barcelona, Spain approved the study.

Note that full information on the approval of the study protocol must also be provided in the manuscript.

Clinical data

Policy information about clinical studies

All manuscripts should comply with the ICMJE guidelines for publication of clinical research and a completed CONSORT checklist must be included with all submissions.

Clinical trial registration	NCT01669252
Study protocol	This is an exploratory study of the clinical study NCT01669252, that soon will be submitted for publication.
Data collection	The stage I-II HER2-negative breast cancer patients reported here were treated at the Vall Hebron Institute of Oncology, Barcelona during the years 2012 and 2013.
Outcomes	The pathological complete response was defined as the absence of residual invasive tumour in breast tumour specimens.
